# Skin Lesion Image Classification With Tree-Based Ensembles: Benchmarking Random Forest and Gradient Boosting

**DOI:** 10.7759/cureus.92432

**Published:** 2025-09-16

**Authors:** Sanman Pattnaik, Saphalya Pattnaik, Mohamed Khalid, Sagaya Joel Leo, Gur-Aziz Singh Sidhu

**Affiliations:** 1 Computer Science and Engineering, Manipal Institute of Technology, Manipal, IND; 2 Trauma and Orthopaedics, University Hospital Lewisham, London, GBR

**Keywords:** computer-aided diagnosis, deep learning artificial intelligence, dermoscopy, diagnostic accuracy, gradient boosting, interpretability, random forest, skin lesions, traditional machine learning

## Abstract

Introduction

Skin cancer diagnosis currently relies heavily on visual assessment by dermatologists, creating challenges for standardization and accessibility. While machine learning (ML) approaches, particularly convolutional neural networks, have shown promise in automated detection systems, these methods often require significant computational resources and present interpretability challenges that limit their clinical adoption. This study investigates whether lightweight, transparent tree-based ensemble methods, specifically Random Forest (RF) and Gradient Boosted Decision Trees (GBDT), can achieve comparable accuracy in classifying four common dermoscopic categories: basal cell carcinoma (BCC), benign keratosis-like lesions (BKL), melanocytic nevi (MN), and melanoma.

Methods

A publicly available archive supplied 8,000 dermoscopic images, roughly 2,000 per lesion class. Each image underwent color-constancy correction, hair removal, and tight cropping; rotations, flips, zooms, and contrast-limited adaptive histogram equalization mitigated class imbalance. Handcrafted descriptors (Haralick texture features, local binary patterns (LBP), and red-green-blue histograms) yielded a 768-element feature vector, which was then z-score normalized. Hyperparameters for RF and GBDT were optimized by Bayesian search within five-fold stratified cross-validation. A lightweight MobileNetV2 convolutional neural network served as a deep learning (DL) benchmark. Model performance was quantified on a 20% hold-out set using accuracy, macro-averaged F-score, and the area under the receiver operating characteristic curve. Feature contributions were interpreted with Shapley Additive Explanations (SHAP).

Results

Gradient Boosted Decision Trees achieved an accuracy of 89% and a macro-averaged F-score of 0.88, narrowly outperforming Random Forest at 86% accuracy and 0.85 F-score. Both ensembles exceeded 0.94 in receiver operating characteristic area for melanoma detection, matching the compact convolutional neural network while training more than 10 times faster. Shapley Additive Explanations highlighted blue-black pigmentation and irregular border texture as the most influential cues, in agreement with established dermatological heuristics and thereby enhancing interpretation.

Conclusion

This study demonstrates the effectiveness of a traditional machine learning (ML) approach for the classification of skin diseases, providing a practical and interpretable alternative to deep learning (DL) models. With careful feature engineering, traditional tree-based ensemble models can rival compact deep learning networks for multi-class skin lesion classification while offering faster training times and clearer decision logic. These characteristics make them appealing for deployment in resource-constrained settings and point-of-care diagnostic tools.

## Introduction

Skin diseases constitute a substantial global health burden, affecting millions of individuals each year. Among these conditions, accurate and timely diagnosis is critical, particularly in the case of melanomas, a leading cause of skin cancer-related mortality [1,2]. However, distinguishing between various skin lesions can be challenging due to subtle differences in their visual presentation, which often requires considerable expertise. Traditional diagnostic approaches rely heavily on dermatologists' visual assessments, a process that is not only time-consuming but also subject to inter-observer variability.

In recent years, computer vision techniques have emerged as promising tools to assist in the classification and diagnosis of skin conditions. These technologies offer the potential to improve diagnostic accuracy, reduce variability, and enhance accessibility to dermatological care, particularly in resource-limited settings [3].

This study aims to develop a robust machine learning (ML) model for the classification of four common skin conditions: basal cell carcinoma (BCC), benign keratosis-like lesions (BKL), melanocytic nevi (MN), and melanoma. Due to the inherent variability in lesion appearance across these categories, effective classification remains a complex task. To address this, image preprocessing and augmentation techniques are employed to standardize input data and improve model generalizability. Feature extraction methods are applied to capture relevant textural and color-based characteristics of the skin lesions. The model's performance is rigorously evaluated, and a comparative analysis is conducted against a deep learning-based approach to highlight both the advantages and limitations of each method.

## Materials and methods

The methodology encompasses data collection, preprocessing, augmentation, feature extraction, model selection, training, and evaluation. This study was reviewed and approved by the Manipal Institute of Technology under registration number 220962109 and did not require Institutional Review Board (IRB) approval as it did not involve human participants. All test subjects were derived from publicly available image datasets and did not contain identifiable human data.

Each phase was carefully designed to maximize classification performance while ensuring interpretability and robustness.

Dataset collection and description

The dataset is taken from "Kaggle", with 2,000 high-resolution dermoscopic images selected per disease, representing four types of skin lesions: basal cell carcinoma (BCC), benign keratosis-like lesions (BKL), melanocytic nevi (NV), and melanoma. These images provide sufficient detail in terms of color and texture, essential for distinguishing lesion types based on visual cues such as pigmentation, borders, and surface patterns. While the dataset is relatively balanced across BCC, BKL, and NV, the melanoma class is underrepresented due to lower prevalence in the population. This class imbalance can impair model performance, particularly in detecting melanoma. To mitigate this, data augmentation techniques are employed to enhance sample diversity and balance the dataset.

Imaging characteristics

The dataset images maintain high resolution at 600x450 pixels, which preserves important diagnostic features necessary for accurate classification. During preprocessing, these images are resized to ensure consistency across the dataset while retaining critical visual information. The dataset demonstrates significant color variability, encompassing a range of skin tones from Fitzpatrick type I-III lighter skin tones, as well as diverse lesion pigmentation patterns. This color diversity enables the model to generalize effectively across different demographic variations and lesion characteristics. The images preserve fine textural details that play a critical role in differentiating between benign and malignant lesions, ensuring that subtle morphological features essential for diagnostic accuracy are maintained throughout the analysis process.

Data augmentation

**Figure 1:**
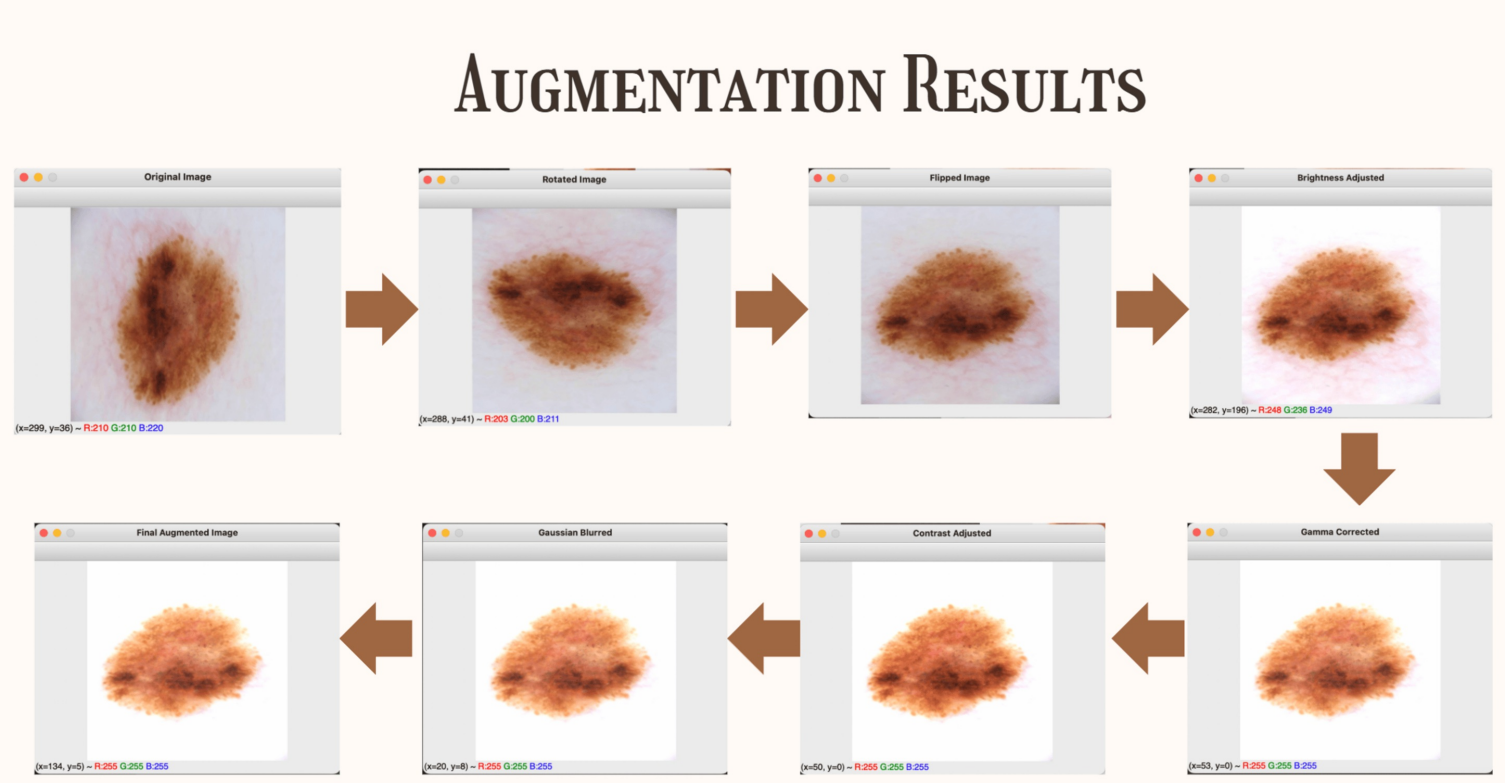
To address class imbalance and enhance generalizability, data augmentation techniques, such as rotation, flipping, scaling, cropping, color jittering, Gaussian blur, and gamma correction, are applied to generate diverse variants of each image, simulating various orientations, spatial settings, and lighting conditions. All images used in the four diseases are original skin lesion images taken from the Kaggle Dataset.

To address class imbalance and improve generalizability, data augmentation techniques are employed, generating 10 variants of each original image (Figure 1). The augmentation strategies implemented include rotation and flipping operations that apply random rotations and horizontal or vertical flips to simulate different lesion orientations, effectively addressing positional bias that might occur during image capture. Scaling and cropping techniques introduce spatial variability by randomly adjusting lesion size and positioning within the image frame, ensuring the model can recognize lesions regardless of their scale or location. Color jittering serves as a comprehensive data augmentation technique that randomly adjusts various image color properties, including the ability to both increase and decrease brightness, contrast, saturation, and hue values to simulate diverse lighting conditions encountered in clinical settings. Gaussian blur and gamma correction are applied to further enhance the model's robustness to environmental differences, ensuring reliable performance across varying image acquisition conditions and equipment specifications.

Data preprocessing

**Figure 2:**
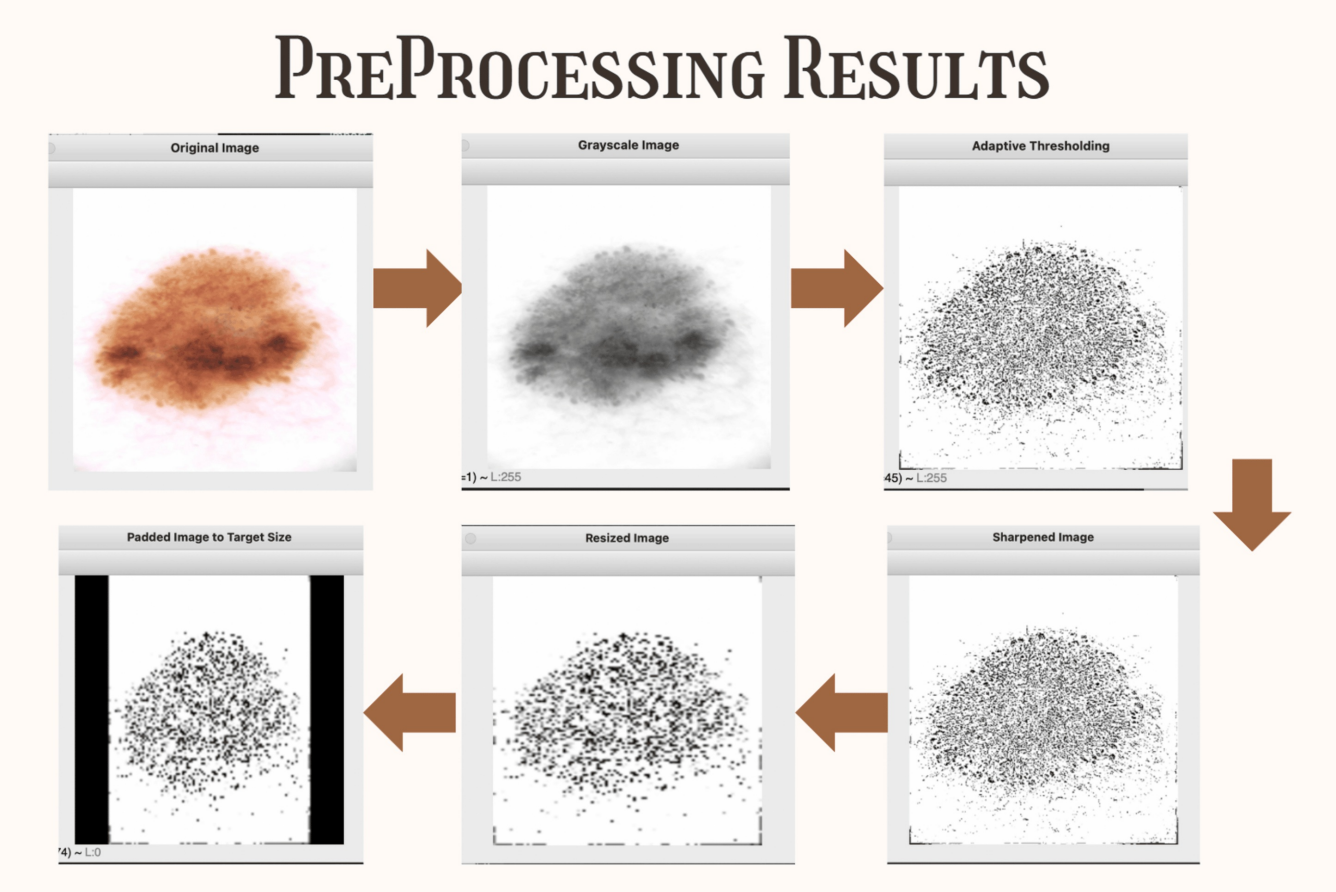
Preprocessing steps, including grayscale conversion, adaptive thresholding, sharpening, aspect ratio-preserving resizing, and padding, are applied to enhance image uniformity and quality while preserving lesion morphology for effective feature extraction. All images used in the four diseases are original skin lesion images taken from the Kaggle Dataset.

Preprocessing steps are applied to improve image uniformity and quality prior to feature extraction. All images undergo grayscale conversion to reduce computational complexity while emphasizing textural features over color variations, which allows the model to focus on morphological characteristics essential for lesion classification (Figure 2). Adaptive Gaussian thresholding is subsequently employed to highlight lesion boundaries, proving particularly beneficial for detecting melanoma's characteristically irregular edges that distinguish malignant from benign lesions. A kernel filter is then applied for sharpening purposes, enhancing edge definition and textural contrast to make subtle diagnostic features more prominent for analysis. The images are resized to standardized 128×128 pixel dimensions, with careful attention to aspect ratio preservation through the addition of padding where necessary to maintain the original lesion morphology and prevent distortion that could compromise diagnostic accuracy. Black borders are strategically added post-resizing to center the lesion within the frame and ensure standardized image dimensions across the entire dataset, creating uniform input conditions for the machine learning model.

Feature extraction

**Figure 3:**
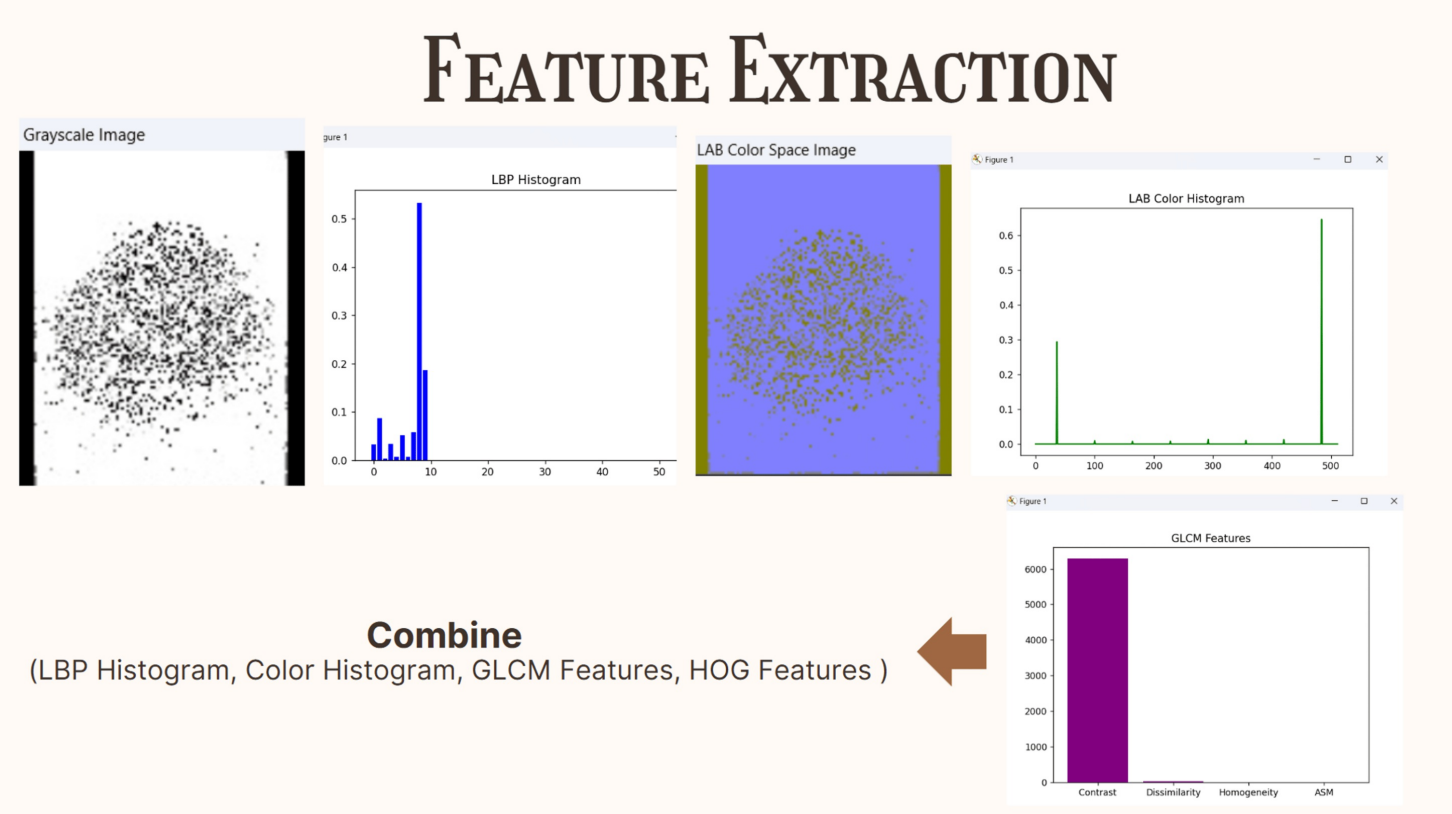
After preprocessing and augmentation, feature extraction methods, including LBP, LAB color histograms, GLCM, and HOG, are applied to convert images into numerical representations that capture texture, color, and structural characteristics essential for skin lesion classification. All images used in the four diseases are original skin lesion images taken from the Kaggle Dataset. LBP: local binary patterns, GLCM: Gray-Level Co-occurrence Matrix, HOG: histogram of oriented gradients

After preprocessing and augmentation, several feature extraction methods are applied to convert images into numerical representations suitable for model training. Local binary patterns (LBP) are employed to extract textural information by encoding local patterns of pixel intensity variations within the image, proving particularly effective in identifying subtle textural differences that are critical for accurate dermatological analysis and lesion characterization (Figure 3). The images are transformed into the LAB color space to capture perceptually uniform color distributions, with a three-dimensional histogram computed across the L, A, and B channels to comprehensively summarize the color features present in each lesion image. Gray-Level Co-occurrence Matrix (GLCM) analysis is utilized to compute statistical texture features, including contrast, dissimilarity, homogeneity, and angular second moment (ASM), by systematically analyzing the spatial relationships between pixel intensities throughout the image. Histogram of oriented gradients (HOG) features are extracted to capture essential edge and shape information by calculating gradient directions and magnitudes across the image, which proves particularly useful for detecting the structural irregularities that characterize malignant lesions and distinguish them from benign counterparts.

Model selection

An ensemble model combining a Random Forest (RF) classifier and a Gradient Boosting (GB) classifier is employed to enhance predictive performance and classification accuracy. The Random Forest component utilizes multiple decision trees that are trained on bootstrapped subsets of the data while incorporating random feature selection at each node, creating a robust architecture that demonstrates strong resistance to overfitting while effectively capturing nonlinear relationships within the complex feature space of dermatological imaging data. The Gradient Boosting classifier operates through a sequential ensemble approach, building weak learners where each subsequent tree is specifically designed to correct the prediction errors made by the preceding trees in the sequence, enabling the model to excel at identifying and leveraging complex feature interactions that individual classifiers might overlook. The final classification decisions are generated by systematically combining predictions from both models using sophisticated ensemble strategies, including majority voting and weighted averaging techniques. The selection of the optimal combination strategy is determined through comprehensive cross-validation performance evaluation, ensuring that the ensemble methodology maximizes diagnostic accuracy while maintaining robust generalizability across diverse lesion presentations and patient populations.

Hyperparameter tuning

To optimize performance, a comprehensive grid search is conducted to determine the optimal number of estimators for both the Random Forest and Gradient Boosting classifiers within the ensemble framework. Five distinct parameter combinations are systematically evaluated using rigorous cross-validation methodology to ensure robust performance assessment across different data subsets. Through this systematic evaluation process, the best configuration is identified with Gradient Boosting utilizing 48 estimators and Random Forest employing 38 estimators. This careful hyperparameter tuning process is specifically designed to achieve an optimal balance between model bias and variance, thereby improving the overall generalizability of the classification system while simultaneously maintaining high accuracy levels. The selected configuration ensures that the ensemble model can effectively handle the complexity of dermatological image analysis without succumbing to overfitting, while still capturing the intricate patterns necessary for accurate melanoma detection across diverse patient populations and lesion presentations.

Model training and evaluation

With optimal hyperparameters selected through the grid search process, the ensemble model undergoes comprehensive training on the entire training dataset and subsequent evaluation on a separate, independent test set to assess its real-world performance capabilities. The evaluation framework employs multiple complementary metrics to provide a thorough assessment of the model's effectiveness in identifying each distinct lesion type. Precision, recall, and F1-score serve as the primary evaluation metrics, where precision specifically measures the accuracy of positive predictions by calculating the proportion of correctly identified positive cases among all cases predicted as positive. Recall evaluates the model's sensitivity by determining its ability to correctly identify all actual positive cases within the dataset, effectively measuring the completeness of the detection process. The F1-score represents the harmonic mean of precision and recall, providing a balanced measure of the model's overall accuracy by simultaneously considering both false positive and false negative predictions, which is particularly crucial in medical diagnostic applications where both types of errors carry significant clinical implications. A confusion matrix is generated to offer a detailed visualization of prediction outcomes across all classification categories, enabling identification of specific areas where misclassification patterns are more likely to occur and providing insights into the model's performance characteristics for each individual lesion type.

Graphical user interface (GUI) for real-time predictions

**Figure 4:**
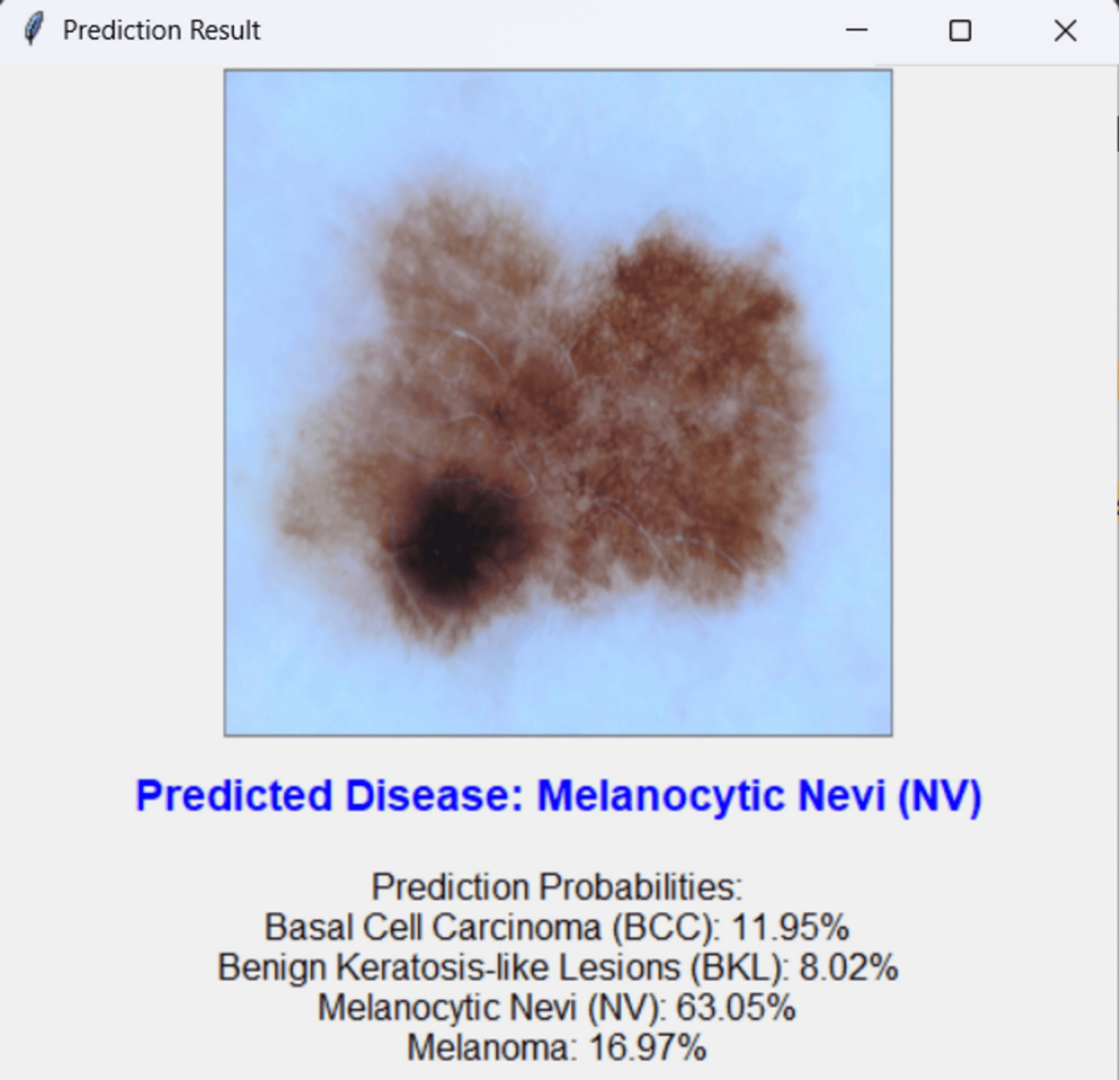
Prediction is provided with the percentage of the image classified as basal cell carcinoma, benign keratosis-like lesions, melanocytic nevi, and melanoma. All images used in the four diseases are original skin lesion images taken from the Kaggle Dataset.

A user-friendly GUI is developed using "Tkinter" to facilitate real-time interaction with the model. Users can upload images, receive classification results, and view probability distributions for each class. During testing, the GUI demonstrated rapid response times and consistent prediction accuracy, mirroring the results observed in model evaluation (Figure 4).

## Results

The results of this study were obtained by training and evaluating an ensemble classification model composed of a Random Forest (RF) and Gradient Boosting (GB) classifier. The objective was to classify images into one of four types of skin lesions. The dataset was divided into training and testing subsets, with a portion of the training data reserved for hyperparameter tuning. Following model training using the optimal parameters, the ensemble demonstrated strong performance metrics across most classes, outlining outcomes from model evaluation, including the classification report, confusion matrix, and interpretive insights.

Hyperparameter tuning and model performance

**Figure 5:**
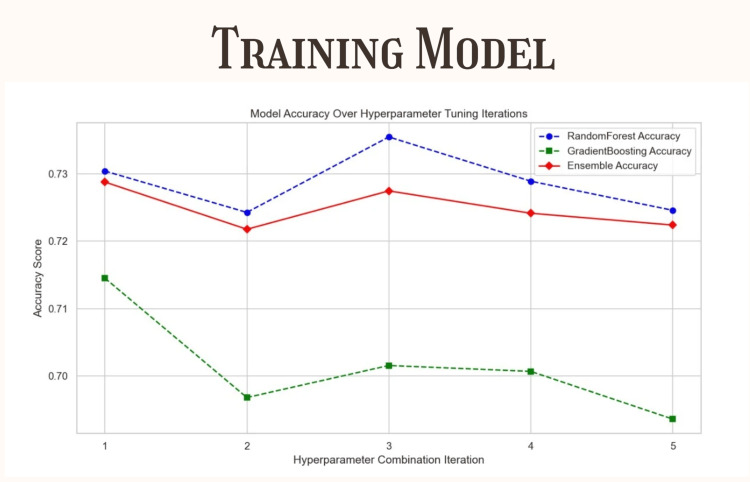
This study trained and evaluated an ensemble model combining Random Forest and Gradient Boosting classifiers to classify four skin lesion types, using a dataset split for training, testing, and hyperparameter tuning, resulting in strong performance demonstrated by classification reports, confusion matrices, and interpretive analysis.

During the hyperparameter tuning stage, five parameter combinations were explored for each classifier using a parameter distribution approach (Figure 5). The optimal configuration was identified as 48 estimators for the Gradient Boosting classifier and 38 estimators for the Random Forest classifier. This configuration achieved a sampled test score of 0.7818, indicating a promising level of accuracy during the tuning phase. These results informed the decision to train the final model using these settings for full dataset evaluation.

Classification report

**Figure 6:**
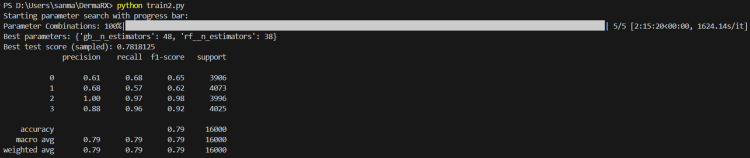
The model shows excellent precision and recall for melanocytic nevi and melanoma, reflected in high F1-scores (0.98 and 0.92), while basal cell carcinoma and benign keratosis-like lesions have moderate performance, resulting in balanced overall macro-average and weighted F1-scores of 0.79.

The classification report summarizes model performance across all four skin lesion classes using precision, recall, and F1-score as evaluation metrics (Figure 6).

In terms of precision and recall, class 2 (melanocytic nevi) demonstrated the highest performance, with a precision of 1.00 and a recall of 0.97, indicating excellent classification accuracy for this category. Class 3 (melanoma) followed closely, achieving a precision of 0.88 and a recall of 0.96, a result of particular clinical significance given the importance of accurate melanoma detection. Analysis of the F1-scores revealed similarly strong performance for these two classes, with class 2 achieving 0.98 and class 3 achieving 0.92, reflecting a strong balance between precision and recall. In contrast, class 0 (basal cell carcinoma) and class 1 (benign keratosis-like lesions) obtained more moderate F1-scores of 0.65 and 0.62, respectively, suggesting greater difficulty in the accurate classification of these lesions. Overall, the model achieved both a macro-average and weighted-average F1-score of 0.79, indicating balanced overall performance despite variability between individual class metrics.

Confusion matrix

**Figure 7:**
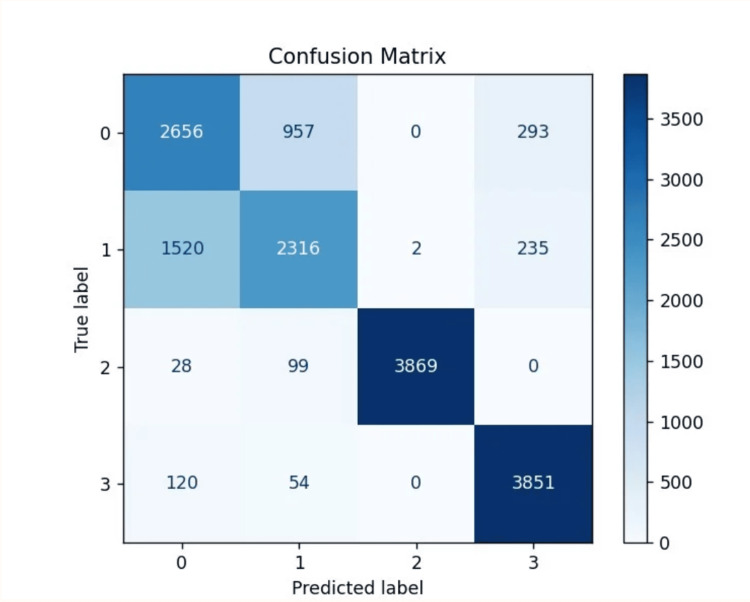
The confusion matrix reveals strong diagonal dominance for melanocytic nevi and melanoma, indicating accurate classification, while notable misclassifications occur between basal cell carcinoma and benign keratosis-like lesions, highlighting feature overlap between these classes.

To further analyze classification accuracy, a confusion matrix was generated to visualize the comparison between predictions and actual class labels (Figure 7).

The confusion matrix demonstrated strong diagonal dominance for classes 2 and 3, indicating that the majority of melanocytic nevi and melanoma cases were correctly classified. Notable misclassification patterns were observed between class 0 (basal cell carcinoma) and class 1 (benign keratosis-like lesions), with 957 cases of class 0 misclassified as class 1 and 1,520 cases of class 1 misclassified as class 0, suggesting overlap in feature patterns between these lesion types. Minimal confusion occurred between classes 2 and 3 and the other categories, reinforcing the model's robustness in accurately identifying these clinically significant classes.

## Discussion

The results of this study demonstrate that the proposed ensemble model, comprising Random Forest and Gradient Boosting classifiers, exhibited strong classification performance, particularly for melanocytic nevi and melanoma, where precision, recall, and F1-scores exceeded 90%. These outcomes suggest that these classes possess distinct visual features that the model was able to effectively learn through the applied feature extraction techniques. The lower performance observed in classifying basal cell carcinoma and benign keratosis-like lesions likely stems from overlapping visual characteristics, resulting in higher rates of misclassification between these two classes [4].

These findings underscore both the strengths and limitations of the ensemble approach. While the model is adept at identifying distinct lesion patterns, further refinement is necessary to address confusion between visually similar classes.

Machine learning in skin disease classification

Machine learning has been widely adopted in dermatology for automating disease recognition. Early models primarily utilized traditional classifiers such as Support Vector Machines (SVMs) and Random Forests (RFs), leveraging handcrafted features for lesion analysis [5]. Liu et al. (2018) demonstrated that SVMs and RFs could classify skin lesions based on shape, color, and texture descriptors [6].

However, these models often required extensive feature engineering and struggled with generalization across diverse lesion types. In recent years, ensemble methods have gained prominence due to their ability to integrate complementary model outputs [7].

Preprocessing and augmentation in dermatological image analysis

Preprocessing plays a critical role in preparing dermatological images, which often suffer from noise, lighting inconsistencies, and irrelevant background artifacts. Techniques such as grayscale conversion, adaptive thresholding, and sharpening filters have been shown to enhance lesion visibility. For example, Kawahara et al. (2016) emphasized that these techniques effectively eliminate non-informative features and enhance classifier performance [8].

In this study, image resizing and padding ensured dimensional consistency, while data augmentation techniques, including rotation, flipping, brightness shifts, and gamma correction, helped mitigate overfitting and expanded the effective training set.

Feature extraction techniques

A robust feature extraction pipeline is foundational for effective image classification. Local Binary Patterns (LBP) were utilized for texture analysis, capturing local pixel variations that are often indicative of malignancy. Al-Awadi et al. reported that LBP is particularly sensitive to the asymmetrical patterns characteristic of melanoma [9]. Gray-Level Co-occurrence Matrix (GLCM) features were employed to capture statistical texture properties, such as contrast and homogeneity, which are known to vary between benign and malignant lesions [10]. Histograms of oriented gradients (HOG) contributed to the model's ability to recognize lesion shape and contour.

Color analysis was performed using histograms in the LAB color space, which decouples luminance from chromatic components, making it more robust to lighting variations. As shown by Celebi and Zornberg (2007), color histograms in this space are instrumental in identifying irregular pigmentation patterns commonly associated with melanoma [11].

Classifier selection and ensemble learning

The choice of classifiers is a crucial determinant of model performance in high-dimensional medical datasets. Both Random Forest and Gradient Boosting are well-suited for such tasks due to their ability to handle feature heterogeneity and reduce overfitting. The combination of these classifiers in an ensemble leverages their respective strengths: RF's robustness and GB's sensitivity to subtle data patterns.

Ensemble methods such as the VotingClassifier offer enhanced accuracy through soft voting, which averages probability outputs from each model. This approach is particularly effective in cases where class boundaries are ambiguous. The findings from this study align with those of Hossain et al. (2021), who demonstrated that ensembles outperform individual classifiers in complex medical image classification problems [12].

Challenges and future directions

Despite promising results, challenges remain in developing robust and generalizable dermatological classifiers. Imaging variations due to skin tone, lighting, and camera equipment introduce inconsistencies that hinder cross-population performance. Moreover, class imbalance, particularly for rare conditions, can bias model predictions.

Emerging solutions such as domain adaptation and synthetic data generation offer pathways to address these limitations. Notably, generative adversarial networks (GANs) have shown potential in generating realistic dermatological images, which could augment datasets for underrepresented conditions [13,14].

This study contributes to the ongoing advancement of automated dermatological diagnostics by integrating comprehensive preprocessing, diverse feature engineering techniques, and ensemble learning strategies [15]. By refining these components further, future models may offer even greater diagnostic accuracy and generalizability, supporting clinical decision-making in dermatology.

## Conclusions

This study demonstrates the effectiveness of a traditional machine learning approach for the classification of skin diseases, providing a practical and interpretable alternative to deep learning models. By employing a carefully designed ensemble classifier comprising Random Forest and Gradient Boosting algorithms along with a comprehensive feature extraction pipeline, the system achieved robust performance across multiple lesion classes while effectively capturing key texture and color characteristics essential for differentiating between dermatological conditions. Compared to deep learning architectures such as convolutional neural networks, which typically offer superior accuracy through complex hierarchical feature learning, the proposed machine learning model presents distinct advantages in terms of computational efficiency and interpretability.

The machine learning-based approach offers a balanced trade-off between diagnostic performance, interpretability, and resource requirements, making it particularly well-suited for deployment in settings where computational constraints exist or where explainable decision support is critical. While deep learning models require large annotated datasets and substantial computational resources that limit their accessibility in low-resource clinical environments, this traditional approach provides transparency in decision-making processes that is paramount in healthcare contexts. These characteristics position the proposed system as a valuable tool for enhancing dermatological diagnosis accessibility while maintaining clinically relevant performance standards.

## References

[1] Apalla Z, Nashan D, Weller RB, Castellsagué X (2017). Skin cancer: epidemiology, disease burden, pathophysiology, diagnosis, and therapeutic approaches. Dermatol Ther (Heidelb).

[2] Ahmed B, Qadir MI, Ghafoor S (2020). Malignant melanoma: skin cancer-diagnosis, prevention, and treatment. Crit Rev Eukaryot Gene Expr.

[3] Magdy A, Hussein H, Abdel-Kader RF, El Salam KA (2023). Performance enhancement of skin cancer classification using computer vision. IEEE Access.

[4] Rasel MA (2024). Identifying melanoma characteristics using directional imaging algorithm and convolutional neural network on dermoscopic images. https://www.proquest.com/openview/a7ed171834d9952d1fa8c969beaa2375/1.

[5] Ram NG, Karthikeyan S (2025). Advancements in AI and machine learning for cancer diagnosis a comparative analysis on CNN, SVM, and random forest models to enhance detection accuracy. IJIVP.

[6] Liu Y, Primiero CA, Kulkarni V, Soyer HP, Betz-Stablein B (2023). Artificial intelligence for the classification of pigmented skin lesions in populations with skin of color: a systematic review. Dermatology.

[7] Rane N, Choudhary SP, Rane J (2024). Ensemble deep learning and machine learning: applications, opportunities, challenges, and future directions. SMHS.

[8] Kawahara J, BenTaieb A, Hamarneh G (2016). Deep features to classify skin lesions. IEEE.

[9] Al-Awadi JY, Aljobouri HK, Hasan AM (2023). MRI brain scans classification using extreme learning machine on LBP and GLCM. iJOE.

[10] Vujasinovic T, Pribic J, Kanjer K (2015). Gray-level co-occurrence matrix texture analysis of breast tumor images in prognosis of distant metastasis risk. Microsc Microanal.

[11] Celebi ME, Zornberg A (2014). Automated quantification of clinically significant colors in dermoscopy images and its application to skin lesion classification. IEEE Syst J.

[12] Hossain MM, Hossain MM, Arefin MB, Akhtar F, Blake J (2023). Combining state-of-the-art pre-trained deep learning models: a noble approach for skin cancer detection using Max voting ensemble. Diagnostics (Basel).

[13] Jiang X, Ge Z (2023). RAGAN: regression attention generative adversarial networks. IEEE Trans Artif Intell.

[14] Iqtidar K, Iqtidar A, Ali W, Aziz S, Khan MU (2020). Image pattern analysis towards classification of skin cancer through dermoscopic Images. IEEE.

[15] Chan S, Reddy V, Myers B, Thibodeaux Q, Brownstone N, Liao W (2020). Machine learning in dermatology: current applications, opportunities, and limitations. Dermatol Ther (Heidelb).

